# Supplementary material for: Non-invasive brain stimulation as treatment for motor impairment in people with Parkinson’s disease: Protocol for an umbrella review
Source: PLoS One. 2024 Jun 13;19(6):e0304717. doi: 10.1371/journal.pone.0304717 (PMC11175533; doi:10.1371/journal.pone.0304717)
Supplement: S1 File — (DOCX) [file pone.0304717.s002.docx]

**Non-invasive brain stimulation as treatment for motor impairment in people with parkinson’s disease: protocol for an umbrella review**

**Search Terms**

((((("parkinson disease"[MeSH Terms] OR ("parkinson"[All Fields] AND "disease"[All Fields]) OR "parkinson disease"[All Fields] OR "parkinsons"[All Fields] OR "parkinson"[All Fields] OR "parkinson s"[All Fields] OR "parkinsonian disorders"[MeSH Terms] OR ("parkinsonian"[All Fields] AND "disorders"[All Fields]) OR "parkinsonian disorders"[All Fields] OR "parkinsonism"[All Fields] OR "parkinsonisms"[All Fields] OR "parkinsons s"[All Fields] OR ("parkinson disease"[MeSH Terms] OR ("parkinson"[All Fields] AND "disease"[All Fields]) OR "parkinson disease"[All Fields] OR "parkinsons"[All Fields] OR "parkinson"[All Fields] OR "parkinson s"[All Fields] OR "parkinsonian disorders"[MeSH Terms] OR ("parkinsonian"[All Fields] AND "disorders"[All Fields]) OR "parkinsonian disorders"[All Fields] OR "parkinsonism"[All Fields] OR "parkinsonisms"[All Fields] OR "parkinsons s"[All Fields]))

AND

("transcranial"[All Fields] OR "transcranially"[All Fields])) OR ("transcranial direct current stimulation"[MeSH Terms] OR ("transcranial"[All Fields] AND "direct"[All Fields] AND "current"[All Fields] OR "stimulation"[All Fields]) OR "transcranial direct current stimulation"[All Fields] OR "tdcs"[All Fields]) OR ("electricity"[MeSH Terms] OR "electricity"[All Fields] OR "electric"[All Fields] OR "electrical"[All Fields] OR "electrically"[All Fields] OR "electrics"[All Fields]) OR ("neurostimulation"[All Fields] OR "neurostimulations"[All Fields] OR "neurostimulator"[All Fields] OR "neurostimulators"[All Fields]) OR “neurmodulation"[All Fields] OR “non-invasive"[All Fields] OR “non-invasive brain stimulation"[All Fields]))

AND

("physical examination"[MeSH Terms] OR ("physical"[All Fields] AND "examination"[All Fields]) OR "physical examination"[All Fields] OR "physical"[All Fields] OR "physically"[All Fields] OR "physicals"[All Fields])) OR ("exercise"[MeSH Terms] OR "exercise"[All Fields] OR "exercises"[All Fields] OR "exercise therapy"[MeSH Terms] OR ("exercise"[All Fields] AND "therapy"[All Fields]) OR "exercise therapy"[All Fields] OR "exercise s"[All Fields] OR "exercised"[All Fields] OR "exerciser"[All Fields] OR "exercisers"[All Fields] OR "exercising"[All Fields]) OR ("strength"[All Fields] OR "strengths"[All Fields]) OR ("balance"[All Fields] OR "balanced"[All Fields] OR "balances"[All Fields] OR "balancing"[All Fields]) OR ("gait"[MeSH Terms] OR "gait"[All Fields]))

AND (meta-analysis[Filter] OR review[Filter] OR systematic review[Filter])

AND (english[Filter])
